# Profiling and Characterization of Volatile Components from Non-Fumigated and Sulfur-Fumigated *Flos Lonicerae Japonicae* Using Comprehensive Two-Dimensional Gas Chromatography Time-of-Flight Mass Spectrometry Coupled with Chemical Group Separation

**DOI:** 10.3390/molecules18021368

**Published:** 2013-01-24

**Authors:** Hao Cai, Gang Cao, Li Li, Xiao Liu, Xiao-Qing Ma, Si-Cong Tu, Ya-Jing Lou, Kun-Ming Qin, Song-Lin Li, Bao-Chang Cai

**Affiliations:** 1 Department of Chinese Materia Medica, College of Pharmacy, Nanjing University of Chinese Medicine, Nanjing 210023, China; 2 Engineering Center of State Ministry of Education for Standardization of Chinese Medicine Processing, Nanjing University of Chinese Medicine, Nanjing 210023, China; 3 National First-Class Key Discipline for Science of Chinese Materia Medica, Nanjing University of Chinese Medicine, Nanjing 210023, China; 4 Research Center of TCM Processing Technology, Zhejiang Chinese Medical University, Hangzhou 310053, China; 5 LECO Corporation Shanghai Representative Office, Shanghai 201900, China; 6 Faculty of Medicine, University of New South Wales, Sydney, NSW 2031, Australia; 7 Department of Pharmaceutical Analysis and Metabolomics, Jiangsu Province Academy of Chinese Medicine, Nanjing 210028, China

**Keywords:** sulfur-fumigation, GC×GC-TOF/MS, volatile compounds, *Flos Lonicerae Japonicae*, quality control

## Abstract

*Flos Lonicerae Japonicae* (FLJ) is a popular herb used for many centuries in Traditional Chinese Medicine as a treatment of fever and inflammation. Non-fumigated processing of FLJ has been the traditional approach used in post-harvest preparation of the commodity for commercial use. However, in recent years, natural drying processing of FLJ has been replaced by sulfur-fumigation for efficiency and pest control. Sulfur-fumigation can induce changes in the volatile compounds of the herb, altering its medicinal properties. A comprehensive two-dimensional gas chromatography time-of-flight mass spectrometry (GC×GC-TOF/MS) method was established for the resolution and determination of volatile components in non-fumigated and sulfur-fumigated FLJ. In this paper, analysis of the volatile oils in non-fumigated and sulfur-fumigated (including lab-prepared sulfur- fumigated and industrial sulfur-fumigated) FLJ was performed using GC×GC-TOF/MS. Seventy-three representative volatile components were identified, including furans, alkalies, acids, aldehydes, ketones, alcohols, terpenes, esters, and others, as the main components of FLJ volatile oils. The proposed method was successfully applied for rapid and accurate quality evaluation of FLJ and its related medicinal materials and preparations.

## 1. Introduction

*Flos Lonicerae Japonicae* (FLJ) is derived from the dried flower buds of *Lonicera japonica* Thunb and is a popular medicinal herb used in Traditional Chinese Medicine (TCM). FLJ is known to exhibit a wide spectrum of biological and pharmacological activities, such as antibacterial, anti-inflammatory, antipyretic, antioxidant, antiviral, and hepato-protective effects [1,2]. As a result, FLJ is widely used as a health-care product or consumed in the form of herbal tea. Furthermore, FLJ contains significant amounts of organic acids, flavonoids, volatile oils, iridoid glycosides and saponins that are considered to be the biologically active components critical in many TCM formulas [3,4].

Traditionally, the roots, flowers and rhizomes used in TCM were dried naturally under the sun. However, in recent decades, this practice has been replaced by sulfur-fumigation, a faster and cheaper method for prevention against insects and mould formation during storage [5]. Typically, this process involves the product being placed in the upper levels of a closed chamber while sulfur powder is burnt at the bottom of the chamber overnight. Sulfur dioxide is then released into the chamber and penetrates the herb. Sulfur-fumigation was recently reported to cause chemical transformation of bioactive components in herbs or its extracts, consequently altering bioactivities, pharmacokinetics, or even the toxicity of TCM [6]. In FLJ, post-harvest processing of the flowering head has traditionally involved natural drying processes. In recent years it has been reported that farmers and wholesalers have replaced this process with sulfur-fumigation. To the best of our knowledge, there has been no investigation into the influence of sulfur-fumigation on volatile components of FLJ.

In the past few years, quality evaluations of FLJ and its preparations have been performed by using many analytical techniques including thin layer chromatography (TLC), gas chromatography (GC), high-performance liquid chromatography (HPLC) and liquid chromatography-mass spectrometry (LC-MS) [7,8,9,10]. However, the previous studies have mainly focused on the components of FLJ, such as organic acids, iridoid glycosides, flavonoids and saponins. It appears that study into the chemical compositions of the essential oils of FLJ has largely been overlooked [11,12]. Characterization of the volatile compounds of FLJ could be used as an indicator of the identity and the quality of FLJ. Furthermore, the volatile organic constituents of FLJ may contribute to some of the pharmacological effects of FLJ extracts. As a typical format of multi-dimensional separation system, comprehensive two-dimensional gas chromatography time-of-flight mass spectrometry (GC×GC-TOF/MS) has become an attractive approach for the analyses of volatile oils in TCM at low concentration in a shorter analytical period [13,14]. GC×GC offers greater peak capacity for a complex sample, which can be achieved by combining a long column as the first dimension with a short column in the second dimension, to spread analytes over a second dimension separation space according to orthogonality considerations. The addition of TOF/MS provides a sensitive detector with full-scan MS capability and a high data density in the second dimension separation space [15,16]. In particular, GC×GC connected to MS with time-of-flight (TOF) analyzer is showing specific advantages in providing accurate mass analysis, resolving power, enhanced selectivity, and high-throughput screening for analysis of complex matrixes such as volatile oils [17,18]. These advantages allow unequivocal identification of ingredients with low quantities, as well as the possibility of quantitation at low concentration levels using extracted ion chromatograms. Up to now, several GC×GC methods have been successfully established for qualitative and quantitative analysis of volatile components in TCM in our laboratory. However, to our knowledge, no strategy has been presented for rapid screening and identification of volatile components from non-fumigated and sulfur-fumigated FLJ using combined techniques of GC×GC separation with TOF/MS approaches.

In this study, an integrated approach using GC×GC-TOF/MS with chemical group separation was established and applied for the resolution and determination of volatile components in non-fumigated and sulfur-fumigated FLJ. GC×GC-TOF/MS was employed to detect the corresponding molecular weight of volatile components. In total, nine groups of volatile components, including furans, alkalies, acids, aldehydes, ketones, alcohols, terpenes, esters and others, were identified for profiling and evaluating the non-fumigated and sulfur-fumigated FLJ samples. This method could be applied to rapidly discriminate sulfur-fumigated FLJ among commercial samples. 

## 2. Results and Discussion

### 2.1. Qualitative Analyses of Non-Fumigated and Sulfur-Fumigated Flos Lonicerae Japonicae Volatile Oils

Based on GC×GC-TOF/MS, 73 representative volatile components with match quality greater than 80% in non-fumigated and sulfur-fumigated FLJ were detected. Generally, the first chromatographic column is non-polar, and the second one is medium-polar. The GC×GC system accomplishes true orthogonal separation due to the changes in polarities of two fixed phases and the linear temperature programming.

The volatile fractions of non-fumigated and sulfur-fumigated FLJ essential oils normally contain several classes of compounds that vary over a wide range of concentrations. The compositions of the volatile fractions obtained from non-fumigated and sulfur-fumigated FLJ using the GC×GC-TOF/MS technique are summarized in Table 1. The volatile fractions are characterized by high percentages of furans, alkalies, acids, aldehydes, ketones, alcohols, terpenes, and esters. These components contribute mainly to the fragrance of non-fumigated and sulfur-fumigated FLJ volatile oils. It should be noted that the peak identification of components is based on NIST08, Adams and Wiley6 mass spectra database libraries. Consequently, the quality of FLJ volatile oils can be assessed by comparing the contents of these compounds. With non-fumigated FLJ samples as a reference, the major portion of volatile components in sulfur-fumigated FLJ was lower than that in non-fumigated ones. After sulfur-fumigation, the components including alkalies and most acids were not found in FLJ. It has been reported that FLJ volatile oils display antibacterial, anti-inflammatory, analgesic, antitumor activities, and also have anti-tussive and anti-asthmatic effects. 

Due to the current gaps in knowledge regarding the active components in FLJ volatile oils, further biological research is required to confirm the results of this study. Thus, it is necessary to control the main volatile target compounds in FLJ through good agricultural practice and traditional processing methods to maintain the quality of Chinese herbal medicines.

### 2.2. Chemical Group Separation of Non-Fumigated and Sulfur-Fumigated Flos Lonicerae Japonicae Volatile Oils

**Figure 1:**
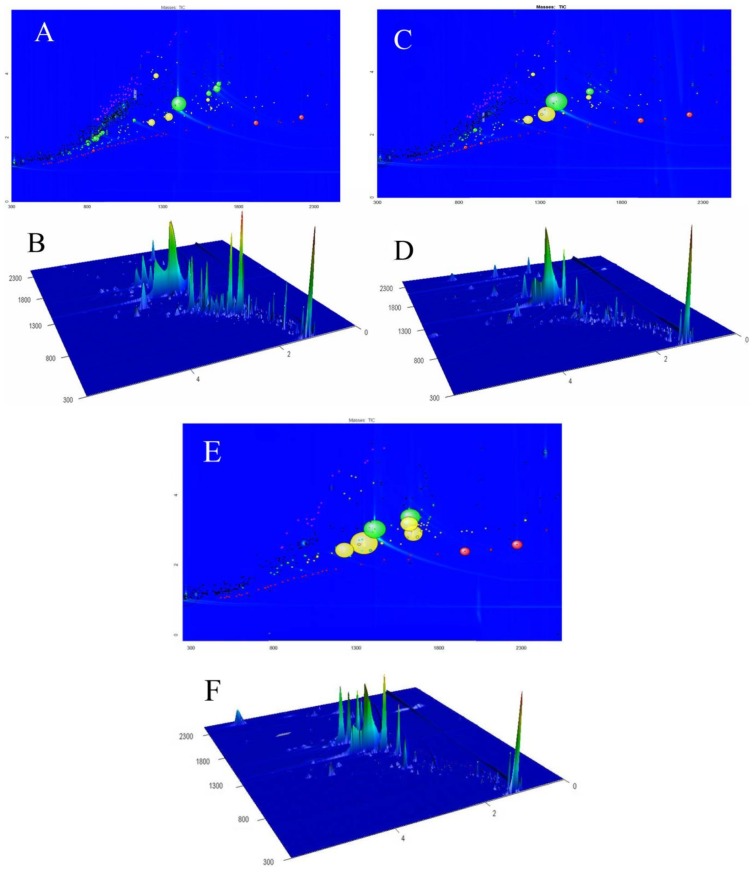
GC×GC-TOF/MS contour plots and three-dimensional chromatograms of non-fumigated (**A**/**B**), lab-prepared sulfur-fumigated (**C**/**D**) and industrial sulfur-fumigated (E/F) *Flos Lonicerae Japonicae* volatile oils. Peak identification information is provided in Table 1.

**Figure 2:**
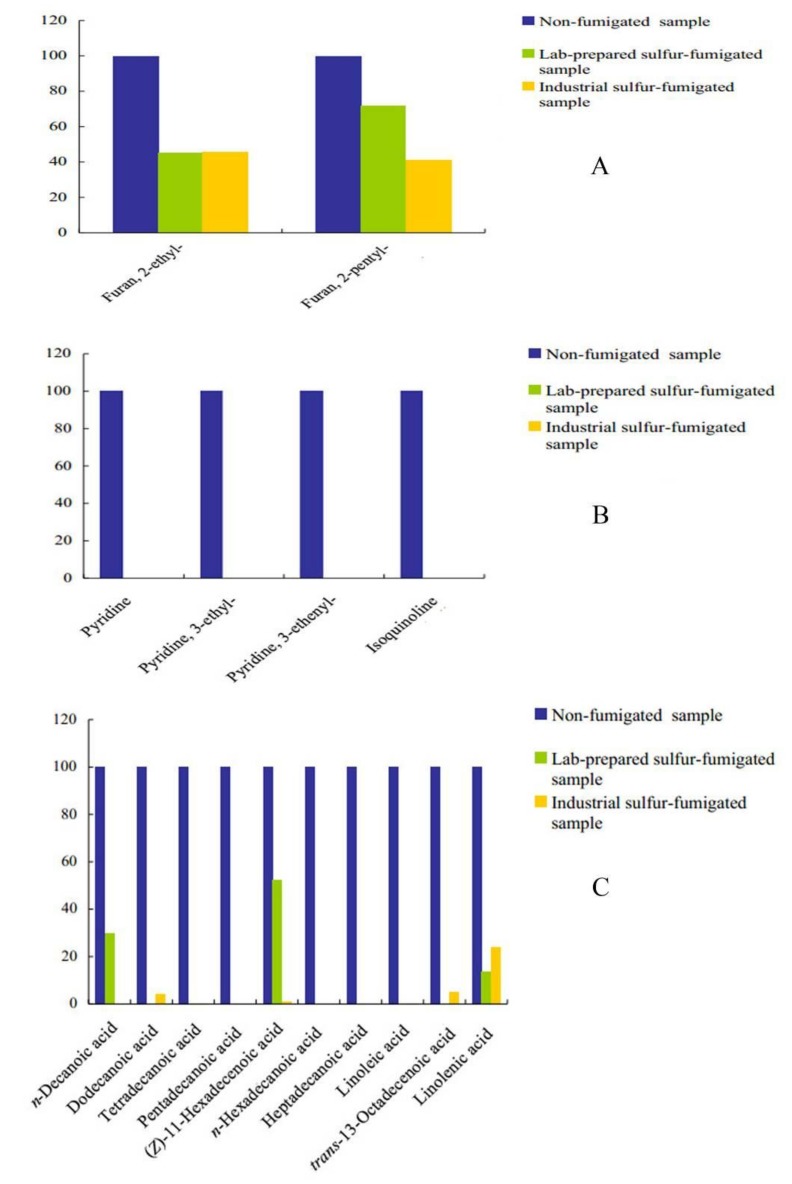

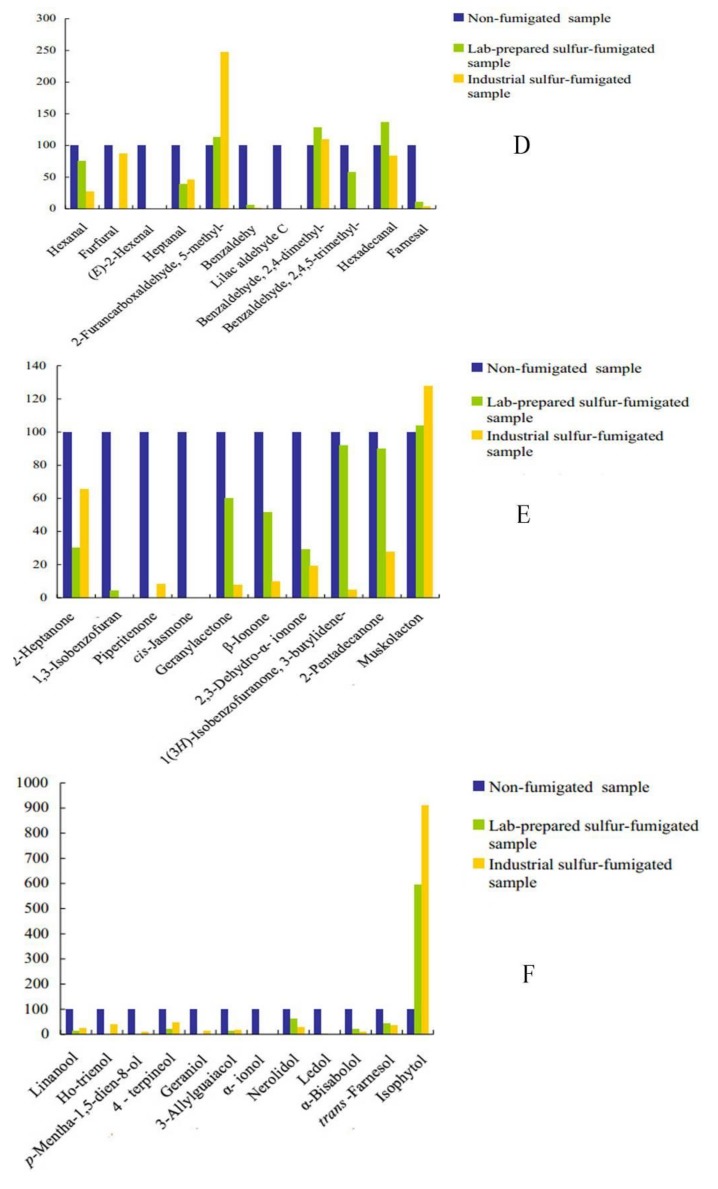

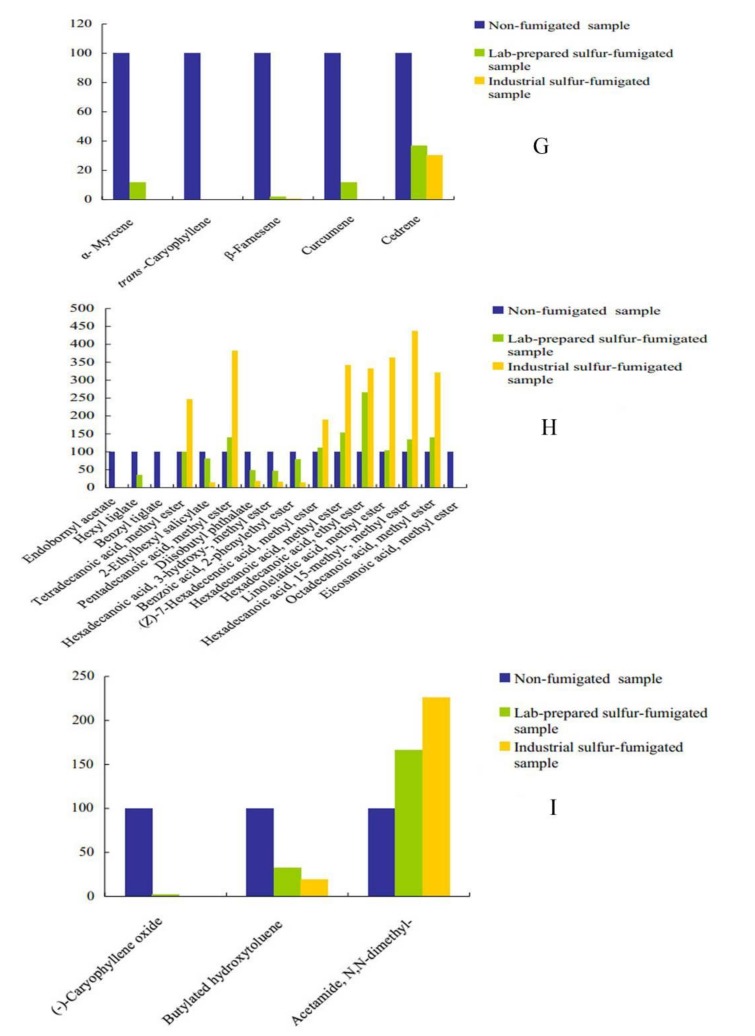
Comparison of the contents of major components in non-fumigated, lab-prepared sulfur-fumigated and industrial sulfur-fumigated *Flos Lonicerae Japonicae* volatile oils. (**A**) Furans, (**B**) Alkalies, (**C**) Acids, (**D**) Aldehydes, (**E**) Ketones, (**F**) Alcohols, (**G**) Terpenes, (**H**) Esters and (**I**) Others.

The column system is orthogonal and provides structured separation. Thus, nine types of components of FLJ volatile oils were detected. The chromatographic peak data consisted of first dimension retention times, second dimension retention times and peak volumes (TIC). The GC×GC chromatogram was constructed as a rasterized image of the TIC computed from each secondary chromatogram (Figure 1). Based on GC×GC-TOF/MS, it can be elucidated that the peaks in the different colored balls are classified for furans, alkalies, acids, aldehydes, ketones, alcohols, terpenes, esters, and others, respectively. The relative content of each component in fumigated sample was compared with non-fumigated sample and the results are shown in Figure 2. It was found that the FLJ volatile oils were constituted by a lot of saturated and unsaturated cyclic hydrocarbons and oxygenated compounds.

### 2.3. Identification of Main Volatile Components in FLJ by GC×GC-TOF/MS

**Figure 3:**
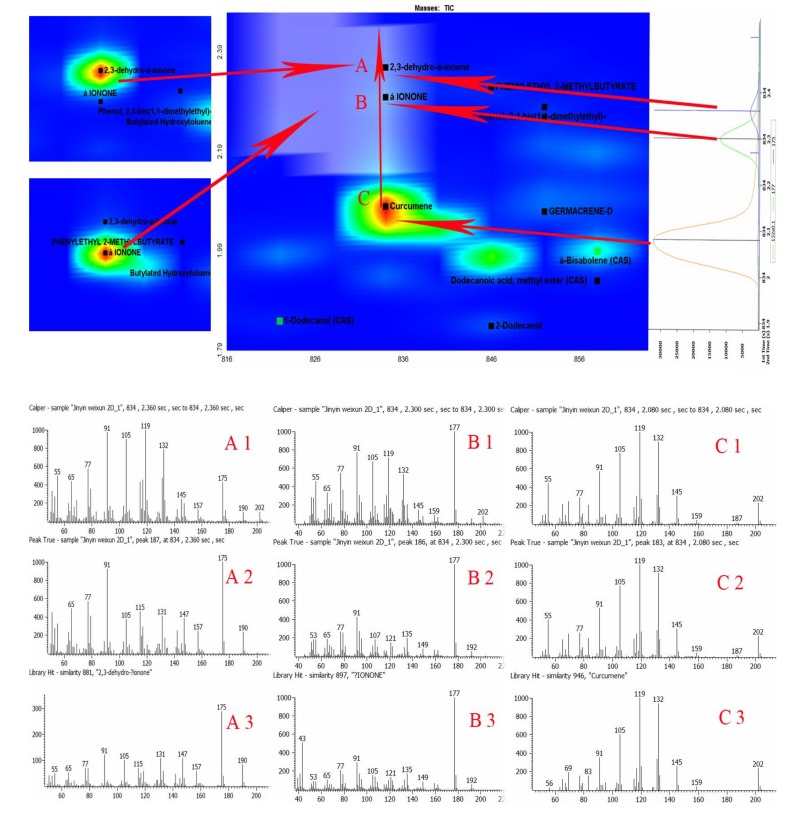
The identified chemical groups of *Flos Lonicerae Japonicae* volatile oil in the GC×GC chromatograms and the spectra of 2,3-dehydro-α-ionone (**A**), α-ionone (**B**) and curcumene (**C**) in sample and in NIST library, respectively (1: Caliper Spectra; 2: Deconvoluted Spectra; 3: NIST Library Spectra).

**Figure 4:**
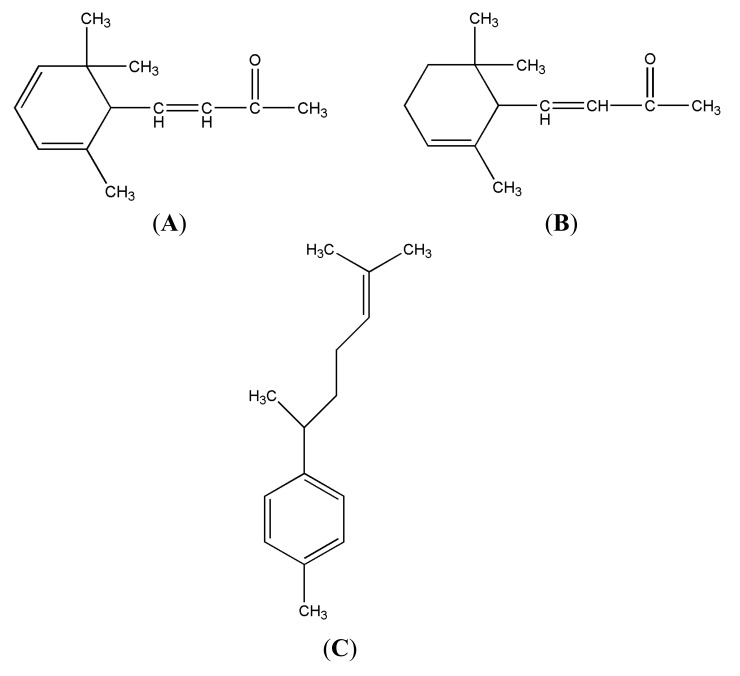
The structures of 2,3-dehydro-α-ionone (**A**), α-ionone (**B**) and curcumene (**C**).

The mass spectra of features of interest in the TIC can be examined to identify compounds, substructures, and elemental compositions. The GC×GC-TOF/MS software was used to determine all the peaks in the raw GC×GC chromatograms. In order to further explain automatic peak search and deconvolution of spectrograms in the software information processing of compounds with common outflow characteristics, sections of the identified chemical groups of FLJ samples were included to elucidate the principle of relative position in the 2D chromatogram as shown in Figure 3. The central portion of the chromatogram showed three compounds, namely curcumene, α-ionone and 2,3-dehydro-α-ionone, with extremely similar RTs. These three compounds overlapped extensively in the 1D GC chromatogram and could be separated by the second dimension column. The Peak Finding algorithm locates the peaks that appear as a single component in the TIC. The Spectral Deconvolution separates the spectra of these overlapping peaks automatically. Good quality spectra could be produced using the deconvolution algorithm, only made possible with TOF. The structures of these three compounds are shown in Figure 4.

## 3. Experimental

### 3.1. Samples and Sample Preparation

Reference FLJ samples were collected from Shandong province and identified by an expert in the field. The lab-prepared sulfur-fumigated samples were prepared from the reference FLJ samples, following procedures similar to that employed by farmers and wholesalers: The reference FLJ samples (250 g) were wetted with water (25 mL), then left standing for 2 h, sulfur powder (25 g) was heated until burning, the burning sulfur and the wetted reference FLJ samples were carefully put into the lower and upper layer of a desiccator, respectively. The desiccator was then kept closed for 6 h. After fumigation, the lab-prepared sulfur-fumigated FLJ samples were dried in a ventilated drying oven at 40 °C for 6 h. Moreover, the industrial sulfur-fumigated FLJ samples, which collected from industrial and commercial process, were also used to investigate compared with the reference FLJ samples. 

The volatile oils of reference and sulfur-fumigated FLJ were extracted using the steam distillation method (Chinese Pharmacopoeia, Eds. 2010) [19]. The volatile oils obtained were dried over anhydrous sodium sulfate (Sigma, St. Louis, MO, USA), then dissolved in ethyl acetate, the concentrations of reference and sulfur-fumigated FLJ were all about 0.2 g/mL, and stored in dark glass bottles at 4 °C until analysis.

### 3.2. GC×GC-TOF/MS Apparatus

A LECO time-of-flight (TOF) mass spectrometer model Pegasus 4D (LECO, St. Joseph, MI, USA) connected to an Agilent 6890N GC was used in GC×GC-TOF/MS experiments. An Agilent 7683B autosampler (Agilent, Palo Alto, CA, USA) injected 1.0 μL of sample at a split ratio of 20:1 at 250 °C through an inlet onto column 1. A column set with a non-polar stationary phase primary column and a medium-polar stationary phase secondary column was used. The first dimension chromatographic column was 30 m × 0.25 mm, 0.25 μm film thickness DB-5ms. The second dimension chromatographic column was 2 m × 0.1 mm, 0.1 μm film thickness DB-17ht. The columns were connected by means of a press-fit connector, and the two columns were installed in two ovens. Column 1’s oven was held at 50 °C for 1 min, then increased to 180 °C at a rate of 15 °C/min and held for 10 min. The temperature was then further increased to 260 °C at a rate of 3 °C/min and held for 3 min. Column 2’s oven was held at 55 °C for 1 min, then increased to 185 °C at a rate of 15 °C/min, and further increased to 265 °C at a rate of 3 °C/min and held for 3 min. Ultra high purity helium (99.9995%) was used as the carrier gas in a constant pressure mode at a flow rate of 1.0 mL/min. Injector temperature was set at 250 °C and split mode was used. The transfer line temperature was 250 °C, ion source temperature was 220 °C, detector voltage was −1850 V, filament bias applied electron ionization voltage at 70 eV, and data bunching was set to give a net acquisition rate of 100 Hz (spectra/s) over the mass range of 45–550 Da. The modulation period was 6 s.

### 3.3. Data Processing

The peaks in the contour plot were integrated and quantified using peak volume. The normalization of peak volume was applied to approximately compare the relative contents of the components due to the lack of standard samples. Data were processed using LECO Pegasus4D software. A S/N threshold of 100 and similarity match threshold of 800 (on the scale of 1–999) was used for peak detection and identification. Identification of compounds was achieved by comparing the experimental (TOF/MS) spectra with NIST08, Adams and Wiley 6 database libraries, and supported by experimentally determined retention index (RI) values, when available. The results of the analyses are located in the peak table. All statistical analyses were conducted using JMP version 7.0.1 (SAS Institute Inc., Cary, NC, USA).

## 4. Conclusions

The present study has described the development of a sensitive and comprehensive method for analyzing volatile compounds found in non-fumigated and sulfur-fumigated *Flos Lonicerae Japonicae* through the use of GC×GC-TOF/MS. This study is first successfully applied to GC×GC-TOF/MS analysis of volatile compounds in sulfur-fumigated *Flos Lonicerae Japonicae*. Compared to the previous studies using one-dimensional GC-MS, GC×GC showed higher resolving power and peak capacity. 73 representative volatile compounds with match quality greater than 80% were identified in non-fumigated and sulfur-fumigated *Flos Lonicerae Japonicae* samples. The established method was successfully applied for the rapid identification of sulfur-fumigated *Flos Lonicerae Japonicae* in commercial FLJ samples. The proposed assay provides an important reference, and can be readily utilized as a suitable method for rapid and accurate quality evaluation of *Flos Lonicerae Japonicae* and related medicinal materials.

## Figures and Tables

**Table 1:** 73 representative volatile components identified in non-fumigated and sulfur-fumigated *Flos Lonicerae Japonicae* by GC×GC-TOF/MS.

Group	Name	R.T. (s)	QuantMasses	Similarity	Non-Fumigated Sample (%)	Sulfur-Fumigated Sample	
						Lab-Prepared (%)	Industrial (%)
Furans	Furan, 2-ethyl-	312, 1.200	81	876	100	45.41	45.60
	Furan, 2-pentyl-	498, 1.330	81	891	100	71.64	41.25
Alkalies	Pyridine	342, 1.400	52	955	100	ND	ND
	Pyridine, 3-ethyl-	480, 1.480	92	943	100	ND	ND
	Pyridine, 3-ethenyl-	486, 1.510	104	898	100	ND	ND
	Isoquinoline	684, 2.090	129	935	100	ND	ND
Acids	*n*-Decanoic acid	732, 1.680	60	920	100	29.81	ND
	Dodecanoic acid	888, 2.120	60	925	100	ND	4.03
	Tetradecanoic acid	1110, 2.560	60	909	100	0.09	0.32
	Pentadecanoic acid	1260, 2.650	60	883	100	ND	ND
	(*Z*)-11-Hexadecenoic acid	1380, 3.020	55	919	100	52.46	0.80
	*n*-Hexadecanoic acid	1404, 3.070	87	935	100	ND	ND
	Heptadecanoic acid	1554, 2.930	73	845	100	ND	ND
	Linoleic acid	1656, 3.540	81	952	100	ND	0.32
	*trans*-13-Octadecenoic acid	1668, 3.270	98	860	100	ND	5.10
	Linolenic acid	1668, 3.690	79	923	100	13.61	24.16
Aldehydes	Hexanal	366, 1.280	56	901	100	75.46	26.89
	Furfural	396, 1.480	96	969	100	ND	87.18
	(*E*)-2-Hexenal	402, 1.370	55	955	100	ND	ND
	Heptanal	438, 1.320	70	916	100	39.42	46.60
	2-Furancarboxaldehyde, 5-methyl-	480, 1.540	110	933	100	113.20	247.31
	Benzaldehyde	486, 1.540	106	971	100	5.63	1.78
	Lilac aldehyde C	600, 1.490	55	931	100	ND	ND
	Benzaldehyde, 2,4-dimethyl-	648, 1.770	133	932	100	128.03	109.54
	Benzaldehyde, 2,4,5-trimethyl-	750, 2.220	147	891	100	57.75	ND
	Hexadecanal	1062, 2.340	82	946	100	136.70	83.14
	Farnesal	1098, 2.920	84	947	100	11.34	3.89
Ketones	2-Heptanone	426, 1.330	58	882	100	30.03	65.47
	1,3-Isobenzofurandione	714, 2.390	76	965	100	4.06	ND
	Piperitenone	732, 2.140	150	907	100	ND	8.42
	*cis*-Jasmone	768, 2.210	79	931	100	ND	ND
	Geranylacetone	798, 2.000	69	950	100	59.93	7.69
	β-Ionone	834, 2.300	177	897	100	51.73	9.90
	2,3-Dehydro-α-ionone	834, 2.360	175	881	100	29.24	19.16
	1(3*H*)-Isobenzofuranone, 3-butylidene-	1038, 3.600	159	953	100	92.08	4.73
	2-Pentadecanone	1044, 2.320	58	943	100	89.67	27.81
	Muskolactone	1380, 3.680	83	913	100	103.80	127.84
Alcohols	Linaool	564, 1.360	71	954	100	12.21	22.83
	Ho-trienol	570, 1.380	82	931	100	ND	38.17
	*p*-Mentha-1,5-dien-8-ol	618, 1.520	59	890	100	ND	9.39
	4-terpineol	624, 1.510	71	927	100	19.66	48.30
	Geraniol	660, 1.590	69	959	100	ND	12.07
	3-Allylguaiacol	738, 2.080	164	951	100	14.37	17.29
	α-ionol	750, 1.830	95	868	100	ND	ND
	Nerolidol	900, 2.170	69	939	100	62.60	29.50
	Ledol	1038, 3.000	71	840	100	3.11	ND
	α-Bisabolol	1044, 2.680	69	929	100	20.02	8.65
	*trans*-Farnesol	1068, 2.770	69	942	100	42.09	35.50
	Isophytol	1374, 2.490	71	935	100	594.57	910.91
Terpenes	α-Myrcene	492, 1.300	93	925	100	11.66	ND
	*trans*-Caryophyllene	798, 1.900	133	953	100	ND	ND
	β-Farnesene	804, 1.810	69	947	100	2.14	0.50
	Curcumene	834, 2.080	132	946	100	11.79	0.33
	Cedrene	1158, 3.100	119	881	100	37.02	30.67
Esters	Endobornyl acetate	690, 1.680	95	957	100	ND	ND
	Hexyl tiglate	708, 1.660	101	925	100	35.61	ND
	Benzyl tiglate	846, 2.600	83	950	100	ND	ND
	Tetradecanoic acid, methyl ester	1068, 2.330	74	930	100	100.46	246.35
	2-Ethylhexyl salicylate	1188, 3.000	120	847	100	81.39	13.35
	Pentadecanoic acid, methyl ester	1200, 2.490	74	884	100	140.60	382.27
	Diisobutyl phthalate	1254, 3.940	149	942	100	47.86	17.20
	Hexadecanoic acid, 3-hydroxy-, methyl ester	1266, 2.890	103	920	100	47.04	16.10
	Benzoic acid, 2-phenylethyl ester	1266, 4.540	104	955	100	79.55	14.85
	(*Z*)-7-Hexadecenoic acid, methyl ester	1326, 2.800	74	865	100	111.59	188.94
	Hexadecanoic acid, methyl ester	1338, 2.660	74	937	100	152.72	341.14
	Hexadecanoic acid, ethyl ester	1440, 2.690	88	902	100	265.86	331.69
	Linolelaidic acid, methyl ester	1596, 3.190	81	935	100	104.25	362.59
	Hexadecanoic acid, 15-methyl-, methyl ester	1644, 2.860	74	908	100	135.10	436.97
	Octadecanoic acid, methyl ester	1956, 2.990	74	870	100	139.39	320.32
	Eicosanoic acid, methyl ester	2256, 3.320	74	919	100	ND	ND
Others	(−)-Caryophyllene oxide	954, 2.610	107	869	100	2.44	0.45
	Butylated hydroxytoluene	852, 2.260	205	861	100	33.22	19.69
	Acetamide, *N*,*N*-dimethyl-	414, 1.530	87	962	100	167.00	225.95

ND: Not detected.
